# Role of FDG-PET/CT in children with fever of unknown origin

**DOI:** 10.1007/s00259-020-04707-z

**Published:** 2020-02-07

**Authors:** Jordy P. Pijl, Thomas C. Kwee, G.E. Legger, Helja J.H. Peters, Wineke Armbrust, E.H. Schölvinck, Andor W.J.M. Glaudemans

**Affiliations:** 1 Medical Imaging Center, Department of Radiology, Nuclear Medicine and Molecular Imaging, University of Groningen, University Medical Center Groningen, Groningen, The Netherlands; 2 Beatrix Children’s Hospital, Department of Pediatric Rheumatology and Immunology, University of Groningen, University Medical Center Groningen, Groningen, The Netherlands; 3 Beatrix Children’s Hospital, Department of Pediatric Infectious Diseases, University of Groningen, University Medical Center Groningen, Groningen, The Netherlands

**Keywords:** Pediatrics, Fever, Unknown, Origin, Inflammation, Infection

## Abstract

**Purpose:**

To determine the role of ^18^F-fluoro-2-deoxy-D-glucose positron emission tomography (FDG-PET)/computed tomography (CT) in children with fever of unknown origin (FUO).

**Methods:**

This retrospective single-center study included 110 children (0–18 years) with FUO who underwent FDG-PET/CT between 2010 and 2019. The diagnostic value of FDG-PET/CT for identifying cause of fever was calculated, treatment modifications after FDG-PET/CT were assessed, and logistic regression analyses were performed to identify clinical and biochemical factors associated with FDG-PET/CT outcome.

**Results:**

In 53 out of 110 patients (48%), FDG-PET/CT identified a (true positive) cause of fever. Endocarditis (11%), systemic juvenile idiopathic arthritis (5%), and inflammatory bowel disorder (5%) were the most common causes of FUO. In 42 patients (38%), no cause of fever was found on FDG-PET/CT. In 58 out of 110 patients (53%), treatment modifications were made after FDG-PET/CT. FDG-PET/CT achieved a sensitivity of 85.5%, specificity of 79.2%, positive predictive value of 84.1%, and negative predictive value of 80.9%. On multivariate logistic regression, C-reactive protein was positively associated with finding a true positive focus of fever on FDG-PET/CT (OR = 1.01 (95% CI 1.00–1.02) per mg/L increase in CRP), while leukocyte count was negatively associated with finding a true positive focus of fever (OR = 0.91 (95% CI 0.85–0.97) per 10^9^ leukocytes/L increase).

**Conclusion:**

FDG-PET/CT is a valuable diagnostic tool in the evaluation of children with FUO, since it may detect a true underlying cause in almost half (48%) of all cases where none was found otherwise. It allows full-body evaluation in patients without disease-specific symptoms on one examination. CRP and leukocyte count were significantly associated with FDG-PET/CT results, which may contribute to a priori assessment on the outcome of FDG-PET/CT. Future research could be aimed at evaluating more patient-specific factors to prospectively estimate the added value of FDG-PET/CT in children with FUO.

## Introduction

Fever is defined as an elevated body temperature of 38.0–38.3 °C or higher (1, 2). It is one of the most common symptoms in children presenting at the hospital. In fact, fever is the chief complaint of children who visit the hospital in 16 to 30% of cases (3–5). The differential diagnosis of fever is broad. The most common cause is infection, with autoimmune disease and malignancy as second and third causes, respectively (6, 7). In approximately 50% of infants up to 3 years old with fever, no cause is found (8). In all children with FUO, no definitive cause of fever is found in 10 to 20% of cases, despite extensive history taking, physical examination, and laboratory testing (9).

There is no widespread consensus on the definition of fever of unknown origin (FUO), and various definitions have been applied throughout the years (2, 10–12). Currently, fever in children is considered FUO after 8 or more days of febrile illness, when a careful and thorough history taking, physical examination, and laboratory workup failed to reveal a probable cause of the fever (7, 9, 13). If these criteria apply, but children have fever for less than 8 days, they are sometimes considered to have “fever without source” (FWS) (7, 9).

In the workup of febrile patients, various diagnostic tests may be used to identify the cause of fever. These investigations often include chest radiography, abdominal ultrasonography, laboratory tests such as C-reactive protein (CRP), leukocyte count, urinalysis, and microbiologic tests. More advanced or invasive investigations may include computed tomography (CT), magnetic resonance imaging (MRI), immunologic or organ-specific laboratory investigations, cytologic punctures, and histologic biopsies.

In adults, ^18^F-fluoro-2-deoxy-D-glucose (FDG) positron emission tomography (PET)/CT is known to be helpful for diagnosing infectious or inflammatory foci in patients with FUO (12, 14–20). FDG-PET/CT is already the first imaging modality in several infectious and inflammatory diseases (21).

In children with FUO however, literature about the value of FDG-PET/CT is scarce. In a study by Jasper et al. (22), the results of FDG-PET/CT in 17 children with FUO were reported, and in a study by Blokhuis et al. (23), 28 FDG-PET/CT scans of children with FUO were described. Because of these relatively low numbers of included patients, the role of FDG-PET/CT in children with FUO remains unestablished.

Therefore, the aim of this study was to assess the value of FDG-PET/CT in finding the cause of fever in a large group of children with FUO and FWS.

## Methods

### Study design and patients

The electronic patient database of the University Medical Center Groningen was searched for all patients aged 0 to 18 years who underwent FDG-PET/CT between 2010 and 2019. All children who underwent FDG-PET/CT for the evaluation of fever without a known cause were potentially eligible for inclusion. Fever was defined as a body temperature of ≥ 38.3 °C. FUO was defined as febrile illness of multiple days, during which careful history taking, physical examination, and laboratory workup did not reveal a cause of fever. Because a minimum threshold of 8 days fever is not used in the clinic to diagnose FUO, children who had fever for multiple consecutive days, but less than 8 days, were also included in this study.

Children who underwent FDG-PET/CT but did not have fever, or had an already established focus of infection, inflammation, or malignancy, were excluded. When children had follow-up FDG-PET/CT scans, only the first FDG-PET/CT scan was included.

### Patient data review

The medical files of all children potentially eligible for inclusion were first reviewed for the inclusion and exclusion criteria. When children were eligible for inclusion, their medical files were further reviewed. Age, gender, medical history, duration of fever, physical examination and history taking, laboratory values (hemoglobin, mean corpuscular volume, thrombocyte count, hematocrit, CRP, leukocyte count and differentiation, erythrocyte sedimentation rate), imaging results and procedures, treatment, final diagnosis, and follow-up data were retrieved from the medical files of all included patients.

### FDG-PET/CT acquisition

All scans were performed using an integrated PET/CT system (Biograph mCT 40 or 64 slice PET/CT, Siemens Medical Systems, Knoxville, TN, USA) with 3 min per bed position. Low-dose unenhanced CT was performed for attenuation correction and anatomic mapping with 100 kV and 30 mAs. Some patients (*n* = 13) underwent concomitant full-dose contrast-enhanced CT with a constant tube potential of 80–120 kV and automatic adjustment of mAs in the *z*-direction. Full-dose CT would be applied when there was a suspicion of inflammation or infection in a certain organ, and low-dose CT would be likely not to offer a resolution high enough to accurately diagnose the disease.

Patients had to fast for at least 6 hours, but some newborns fasted for only 4 h because of risk of hypoglycemia. Patients with a (slight) suspicion of a cardiac focus of fever also had to adhere to a diet low in carbohydrates the day before FDG-PET/CT was performed. For example, this diet allowed eating meat, fish, eggs, vegetables, and clear soup, but did not allow potatoes, rice, bread, or milk.

After blood glucose concentrations were ensured to be below 11 mmol/L, 3 MBq/kg FDG was administered intravenously. PET/CT imaging was performed 60 min after FDG administration. Data acquisition and reconstruction were in accordance with EANM/EARL (European Association of Nuclear Medicine/ResEARch 4 Life) guidelines (21, 24). Some of the children had to be sedated or anesthetized when FDG-PET/CT was performed to prevent excessive movement during the scan. Scans were performed from skull to mid-thigh or to toes based on the presence or absence of complaints in the lower extremities.

### Interpretation of FDG-PET/CT results

All FDG-PET/CT scans were prospectively interpreted by nuclear medicine physicians as part of routine clinical care, using Syngo.via software (Siemens Healthineers, Erlangen, Germany). All scans with inconclusive findings (not clear-cut positive or negative for a cause of fever) were re-evaluated by another nuclear medicine physician (AWJM) who was blinded to original FDG-PET/CT interpretations, other imaging results, clinical, laboratory, and microbiologic tests.

### Diagnostic reference standard

The final diagnosis at hospital discharge was used as reference standard for FDG-PET/CT results. This diagnosis was based on results from all examinations performed during hospital admission (including clinical examination, laboratory analysis, microbiologic cultures, histologic biopsies, imaging results), response to treatment, and clinical or outpatient follow-up. FDG-PET/CT scans that did not identify a cause of fever were classified as true negative if patients were diagnosed with FUO at hospital discharge and fever had resolved spontaneously, without a definitive cause of fever found on any additional testing during hospital stay or during follow-up of at least 3 months. Some children with FUO died because of other underlying diseases. Their fever sometimes did not resolve, but their FDG-PET/CT scans could still be considered true negative if their cause of death was another disease not causing the fever. FDG-PET/CT scans were regarded as true positive when the cause of fever found on FDG-PET/CT corresponded with the diagnosis at hospital discharge.

## Statistical analysis

Continuous variables were checked for normal distribution using Kolmogorov-Smirnov tests. Normally distributed data were presented as mean ± standard deviation, and non-normally distributed data as median with interquartile range (IQR). Sensitivity, specificity, positive predictive value (PPV), and negative predictive value (NPV) of FDG-PET/CT for identifying a cause of fever in children with FUO were calculated, along with 95% confidence intervals (CIs).

Age, gender, medical history, duration of fever, laboratory values (CRP, leukocyte count), and CT type (low-dose unenhanced or full-dose contrast-enhanced) were analyzed with univariate logistic regression as independent variables and FDG-PET/CT outcome as dependent variable. The dependent variable was either categorized as true positive or not true positive (i.e., false positive, false negative, or true negative). Corresponding odds ratios (ORs) and 95% CIs were calculated, and *P* < 0.05 was considered statistically significant. Variables with *P* ≤ 0.10 on univariate analysis were included in the stepwise multivariate logistic regression model. All statistical analyses were performed using IBM Statistical Package for the Social Sciences (SPSS) version 26 (SPSS, Chicago, IL, USA).

## Results

### Patient characteristics

**Fig. 1:**
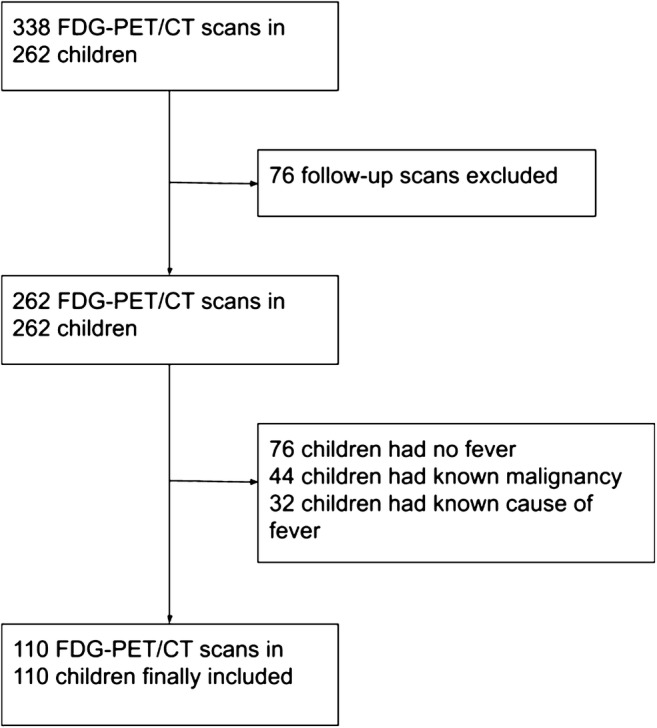
After excluding follow-up FDG-PET/CT scans and excluding patients without fever, with a known malignancy, or any other known cause of fever at the time FDG-PET/CT was performed, 110 FDG-PET/CT scans in 101 patients with FUO and 9 patients with FWS were included

**Fig. 2:**
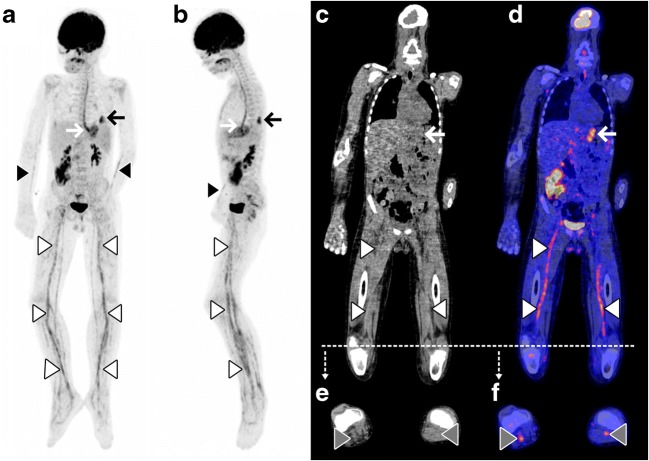
A 9-year-old boy was admitted to the hospital with general malaise present for 10 days, high fever (39.5 °C), painful legs, and bloody diarrhea. Physical examination showed increased respiratory effort, hepatomegaly, ascites, and erythema on the hands and feet. Laboratory testing showed a CRP of 307 mg/L, leukocyte count of 24.3 × 109/L, and erythrocyte sedimentation rate of 55 mm/h. MRI of the head and neck, chest radiography, and abdominal ultrasonography did not reveal any cause of fever, and microbiological cultures were negative for infection. As the patient did not respond to antibiotic treatment, corticosteroid treatment was also started. The symptoms improved, but the exact cause of the symptoms remained unknown, and FDG-PET/CT was performed. Coronal and sagittal maximum intensity projection FDG-PET (A and B), low-dose CT (C) and fused FDG-PET/CT (D), axial low-dose CT (E), and axial fused FDG-PET/CT (F) showed increased FDG uptake in the major arteries of the arms (black arrowheads) and legs (white arrowheads), including the popliteal artery in both knees (gray arrowheads). Based on these findings, the patient was diagnosed with polyarteritis nodosa. As collateral findings, increased FDG uptake in the esophagus due to irritation of a nasogastric feeding tube (white arrow) and a small infectious process in the lower lobe of the left lung (black arrow) were found.

**Fig. 3:**
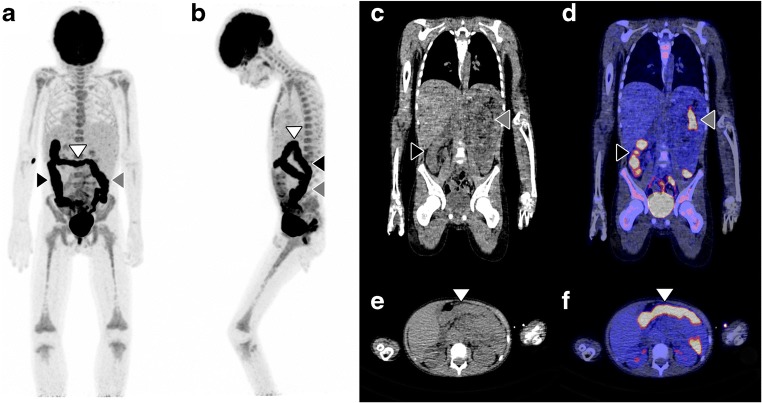
A 9-year-old boy presented at the hospital with anorexia, weight loss, fatigue, and intermittent fever up to 39.5 °C. The patient had experienced these symptoms episodically over the past 18 months, but a definite diagnosis had never been established. On physical examination, the upper abdomen was tender to palpation. Laboratory examinations showed a CRP of 49 mg/L, leukocyte count of 8.0 × 109/L, and erythrocyte sedimentation rate of 46 mm/h. Microbiological urine, blood, and fecal cultures were negative. Abdominal ultrasonography and MRI showed focal steatosis of the liver, but no possible cause of fever. Coronal and sagittal maximum intensity projection FDG-PET (A and B), low-dose CT (C) and fused FDG-PET/CT (D), axial low-dose CT (E), and axial fused FDG-PET/CT (F) showed extensive FDG uptake throughout the entire colon, including the ascending (black arrowheads), transverse (white arrowheads), and descending colon (gray arrowheads). Inflammatory bowel disorder was suspected, and intestinal biopsy established the final diagnosis of Crohn’s disease.

338 FDG-PET/CT scans in 262 children were potentially eligible for inclusion. After excluding follow-up FDG-PET/CT scans and excluding patients without fever, with a known malignancy, or any other known cause of fever at the time FDG-PET/CT was performed, 110 FDG-PET/CT scans in 101 patients with FUO and 9 patients with FWS were included (Fig. 1). The 9 children with FWS had fever for at least 5 days before FDG-PET/CT. The FDG-PET/CT images of two interesting patients with FUO are shown in Figs. 2 and 3. Fifty-four boys and 56 girls were included, with a median age of 9 years. Twenty-four percent of them had no previous medical history, 6% had received an organ or bone marrow transplant, and 10% were taking immunosuppressive drugs before FDG-PET/CT. The median duration of febrile illness before FDG-PET/CT was 25 days. Seven percent of the patients died within 3 months after FDG-PET/CT (Table 1). In 65% of patients, chest radiography was performed before FDG-PET/CT. Fifty-five percent had an abdominal ultrasound, and 11% had an abdominal CT before FDG-PET/CT. In 9% of patients, no previous diagnostic imaging was performed before FDG-PET/CT. Imaging and additional laboratory tests performed before FDG-PET/CT are summarized in Table 2.

**Table 1:** Clinical characteristics of included patients (*n* = 110)

Characteristic	Value
Age (years)	9.0 (12.0)^a^
0–5 years	42
6–11 years	24
12–18 years	44
Number of boys:girls	54:56
Previous medical history	
No medical history	26
Previous episode of unexplained fever	6
Hepatobiliary disease	22
Recurrent ear/nose/throat infections	7
Cardiac abnormality	15
Chromosomal abnormality	8
Other	26
Transplant recipient	
Liver transplant	4
Bone marrow transplant	2
Heart transplant	1
Immunocompromised	
Taking immunosuppressants	11
Duration of febrile illness before FDG-PET/CT (days)	25 (31)^a^
Mortality within 3 months after FDG-PET/CT	8^b^
FDG-PET/CT scan from head to	
Proximal femur	59
Knee	8
Toes	43
FDG-PET/CT characteristics	
Low-dose unenhanced CT	110
Full-dose contrast-enhanced CT	13
Blood glucose level before scan (mmol/L)	4.9 (1.0)^a^
Radioactive dose used (MBq)	86 (101)^a^

^a^Median (interquartile range)

^b^Causes of death: (1) liver and kidney failure, (2) thrombotic liver disease, (3) hepatic encephalopathy, (4) bone marrow failure due to mitochondrial myopathy and sideroblastic anemia, (5) cardiac shunt thrombosis, (6) massive pulmonary bleeding, (7) hemophagocytic lymphohistiocytosis, (8) hepatopulmonary syndrome

**Table 2:** Imaging and laboratory tests before FDG-PET/CT

Test	Value
X-ray performed in number of patients	
Chest	71
Abdomen	24
Extremity	17
Ultrasonography performed in number of patients	
Head/neck	7
Thorax (cardiac)	50
Abdomen	60
Extremity	12
Doppler	18
CT performed in number of patients	
Head/neck	4
Thorax	8
Abdomen	12
MRI performed in number of patients	
Head/neck	7
Abdomen	5
Extremity	5
White blood cell count (× 10^9^/L)	9.8 (8.5)^a^
Neutrophilic granulocytes (× 10^9^/L)	5.6 (7.0)^a^
Lymphocytes (× 10^9^/L)	1.9 (1.4)^a^
Eosinophils (× 10^9^/L)	0.15 (0.27)^a^
C-reactive protein (mg/L)	49 (75)^a^
Hemoglobin (mmol/L)	6.2 ± 1.1^b^
Hematocrit (L/L)	0.30 ± 0.055^b^
Mean corpuscular volume (fL)	81.7 ± 6.3^b^
Erythrocyte sedimentation rate (mm/h)	61 ± 39^b^
Thrombocytes (10^9/L)	309 (254)^a^

^a^Median (interquartile range)

^b^Mean (standard deviation)

### Definitive causes of fever

In 68 out of all 110 patients, a definite cause of fever was found (62%). These included endocarditis in 12 patients, systemic juvenile idiopathic arthritis in 5 patients, inflammatory bowel disorder in 5 patients, and cholangitis in 4 patients (Table 3). In 42 patients (38%), no cause of fever was found on FDG-PET/CT or any other diagnostic test; their diagnoses remained FUO (Table 3).

**Table 3:** Discharge diagnosis after FDG-PET/CT

Diagnosis occurring multiple times	Number of children	Found on FDG-PET/CT	Diagnosis occurring once	Number of children	Found on FDG-PET/CT
Fever of unknown origin	42	n/a	Acute lymphoblastic leukemia	1	1 (100%)
Endocarditis	12	7 (58%)	Hepatic aspergillus infection	1	1 (100%)
Systemic juvenile idiopathic arthritis	5	3 (60%)	Castleman disease	1	1 (100%)
Inflammatory bowel disorder	5	5 (100%)	Ewing sarcoma	1	1 (100%)
Cholangitis	4	4 (100%)	Familial Mediterranean fever	1	n/a
Post-transplant lymphoproliferative disease	3	3 (100%)	Intravenous catheter infection	1	1 (100%)
Pulmonary infection	3	3 (100%)	Mixed connective tissue disorder	1	1 (100%)
Abdominal abscess	2	2 (100%)	Parapharyngeal abscess	1	1 (100%)
Single joint arthritis	2	1 (50%)	Polyarteritis nodosa	1	1 (100%)
Drug-induced fever	2	n/a	Sacroiliitis	1	1 (100%)
Extrapulmonary tuberculosis	2	2 (100%)	Sickle cell crisis	1	1 (100%)
Juvenile idiopathic arthritis	2	2 (100%)	Systemic lupus erythematosus	1	0 (0%)
Kawasaki arteritis	2	n/a	Soft tissue infection leg	1	1 (100%)
Mediastinitis	2	2 (100%)	Tonsillitis	1	1 (100%)
Osteomyelitis	2	2 (100%)	Urinary tract infection	1	n/a
Spondylodiscitis	2	2 (100%)	Visceral leishmaniasis	1	1 (100%)
Takayasu arteritis	2	2 (100%)			

In 53 out of the 68 patients with a confirmed cause of fever, the cause of fever was established based on FDG-PET/CT (Table 3).

In 15 patients, a cause of fever was found that was not diagnosed by FDG-PET/CT. These included 5 cases of endocarditis (of whom 4 patients had an artificial valve), 2 cases of systemic juvenile idiopathic arthritis, 1 single joint arthritis, 2 cases of drug-induced fever, 2 cases of Kawasaki arteritis, 1 case of systemic lupus erythematosus, 1 urinary tract infection, and 1 case of familial Mediterranean fever.

Because drug-induced fever, Kawasaki arteritis, familial Mediterranean fever, and urinary tract infection are not to be diagnosed with FDG-PET/CT, these six cases were not considered “false negative,” but in the absence of other signs of inflammation, malignancy, or infection, as “true negative.”

### Diagnostic performance of FDG-PET/CT

According to the reference standard, 53 FDG-PET/CT results were true positive, 10 were false positive, 38 were true negative, and 9 were false negative. This resulted in a sensitivity of 85.5%, specificity of 79.2%, PPV of 84.1%, and NPV of 80.9% (Table 4). Among the 10 false positive results, 3 FDG-PET/CT scans were suggestive of lymphoma, 3 scans suggestive of pulmonary infection, 2 scans suggestive of inflammatory bowel disorder, 1 scan indicated a soft tissue infection of the leg, and 1 scan was suggestive of vasculitis. The 9 false negative results included five cases of endocarditis, two cases of systemic juvenile idiopathic arthritis, one case of systemic lupus erythematosus, and one single joint arthritis.

**Table 4:** Sensitivity and specificity of FDG-PET/CT

Statistic	Value	95% CI
Sensitivity	85.5% (53/62)	74.2–93.1%
Specificity	79.2% (38/48)	65.0–89.5%
Positive predictive value	84.1%	75.2–90.3%
Negative predictive value	80.9%	69.4–88.7%

### Treatment modifications

In 58 out of 110 patients (53%), treatment modifications were made after FDG-PET/CT. The most common changes included a switch in antibiotics (21 patients), starting immunosuppressive therapy (10 patients), and starting treatment with a non-steroidal anti-inflammatory drug (5 patients). The same treatment as before FDG-PET/CT was continued in 49 out of 110 patients (45%). In 3 patients, it was not documented what treatment was given after FDG-PET/CT (Table 5).

**Table 5:** Changes in treatment after FDG-PET/CT

Treatment change	Number of patients
New treatment started	
Immunosuppressant	10
NSAID	5
Antibiotics	4
Antifungal	4
Chemotherapy	2
Treatment switched	
Antibiotics	21
Immunosuppressant	4
NSAID	2
Treatment stopped	
Antibiotics	4
Unclear	3
Other	2
No change	49

### Factors associated with FDG-PET/CT outcome

On univariate logistic regression, leukocyte count was negatively associated with finding a true positive focus of fever on FDG-PET/CT (OR = 0.93 (95% CI 0.88–0.99) per 10^9^ leukocytes/L increase), and CRP was positively associated with finding a true positive focus (OR = 1.01 (95% CI 1.00–1.01) per mg/L increase in CRP). On multivariate logistic regression, CRP was positively associated with finding a true positive focus of fever on FDG-PET/CT (OR = 1.01 (95% CI 1.00–1.02) per mg/L increase in CRP), while leukocyte count was negatively associated with finding a true positive focus of fever (OR = 0.91 (95% CI 0.85–0.97) per 10^9^ leucocytes/L increase). No other clinical or laboratory factors were significantly associated with FDG-PET/CT outcome (Table 6).

**Table 6:** Factors associated with finding a true positive focus of fever on FDG-PET/CT

Parameter	Univariate OR (95% CI)	*P*	Multivariate OR (95% CI)	*P*
Age	1.00 (0.94–1.06)	0.96		
Gender	0.75 (0.35–1.59)	0.45		
Duration of fever (before FDG-PET/CT)	1.00 (1.00–1.00)	0.50		
CRP	1.01 (1.00–1.01)	0.082	1.01 (1.00–1.02)	0.013
Leukocytes	0.93 (0.88–0.99)	0.019	0.91 (0.85–0.97)	0.004
PET with full-dose contrast-enhanced CT	1.29 (0.41–4.13)	0.66		
Medical history				
Healthy	0.73 (0.30–1.78)	0.49		
Previous episode of unexplained fever	2.25 (0.39–12.80)	0.36		
Hepatobiliary disease	0.87 (0.34–2.23)	0.78		
Recurrent ear/nose/throat infections	0.80 (0.17–3.73)	0.77		
Cardiac abnormality	2.42 (0.77–7.62)	0.13		
Chromosomal abnormality	1.08 (0.26–4.56)	0.92		
Other	0.73 (0.30–1.78)	0.49		

## Discussion

This study shows that FDG-PET/CT can play a valuable role in identifying a cause of fever in children with FUO.

In 68 of all 110 patients with fever (62%), a definite cause of fever was identified. This cause was identified on FDG-PET/CT in 53 of all 110 patients (48%).

Previous research on the value of FDG-PET/CT in children with FUO is limited. In a study by Jasper et al., FDG-PET/CT was performed in 17 children with FUO. They reported a sensitivity of FDG-PET/CT of 100% for finding a focus of fever, but a specificity was not reported (22). It was not specified which patients were included in the sensitivity and specificity analyses, although 43% of included FDG-PET/CT scans were performed for finding an inflammatory focus in patients without FUO. Eighteen percent of FDG-PET/CT results were considered helpful because they excluded differential diagnoses, 24% were considered helpful because they allowed targeted evaluation, and 59% were considered not helpful.

In a study by Blokhuis et al., FDG-PET/CT was performed in 28 children. An infection was found in 7% of patients, malignant disease in 7% of patients, non-infectious inflammatory disease in 32% of patients, and in 54% of patients, no cause of fever was found. Blokhuis et al. reported a sensitivity of 80% and specificity of 78% for finding a cause of fever on FDG-PET/CT (23). A definitive cause of FUO was established in 16 patients, but this cause was found on only 8 FDG-PET/CT scans and 2 FDG-PET scans.

In our patient population, FDG-PET/CT achieved a sensitivity of 85.5% and specificity of 79.2% for identifying a cause of fever. Three out of 10 false positive FDG-PET/CT results were suggestive of lymphoma. FDG-PET/CT has a high sensitivity for diagnosing lymphoproliferative diseases such as lymphoma, but low specificity. As lymphadenopathy is a relatively common finding in patients with FUO, the use of FDG-PET/CT to distinguish benign or reactive from malignant lymphadenopathy is challenging (25, 26). However, FDG-PET/CT is an excellent tool to identify an easily accessible FDG avid lymphoid lesion for diagnostic biopsy.

In 5 out of 9 false negative results, the diagnosis at hospital discharge was endocarditis that was not identified on FDG-PET/CT. Four patients had artificial valve endocarditis, and 1 patient had native valve endocarditis. FDG-PET/CT is known to have a low sensitivity for native valve endocarditis, but a very high sensitivity for artificial valve endocarditis (27). In 2 out of 4 cases of artificial valve endocarditis, the diagnosis was based on clinical signs and positive blood cultures. However, these two patients had not followed a diet low in carbohydrates to suppress physiologic FDG uptake of the myocardium, which may have masked pathologic FDG uptake of the cardiac valves.

As is illustrated by these false negative results, it is important to keep in mind that not all cases of FUO can be diagnosed with FDG-PET/CT. Because of various reasons, the underlying cause of fever may not be visible on FDG-PET/CT. Several precautions can be taken to avoid unnecessary false negative results. Most importantly, these include adhering to a diet low in carbohydrates, especially when a focus of fever is suspected in tissues with high metabolic activity such as the heart, and reducing dosage of corticosteroid treatment to a minimum, especially when vasculitis is suspected. Likewise, prolonged use of antibiotic treatment may reduce the chance of finding a focus of infection (28). When FDG-PET/CT is unable to detect a cause of fever, but there is still clinical suspicion of a certain disease, additional imaging or testing is still warranted.

In 53% of patients, treatment was modified after FDG-PET/CT. Because of the retrospective nature of this study, it is difficult to relate treatment modifications directly to FDG-PET/CT outcome, especially with regard to changes within the same class of medication (e.g., antibiotics and anti-inflammatory agents). However, in 16 out of 53 patients with a true positive cause of fever, a new type of treatment was started, while this was only the case in 4 out of 38 true negative patients. Thus, a new type of treatment was three times more likely to be started in patients with a true positive cause of fever on FDG-PET/CT than in patients with true negative FDG-PET/CT results.

On univariate logistic regression, only leukocyte count was significantly associated with FDG-PET/CT outcome. On multivariate logistic regression, CRP level (positively) and leukocyte count (negatively) were significantly associated with FDG-PET/CT outcome (OR of 1.01 and 0.91 per unit increase, respectively). Although CRP and leukocyte count are sometimes regarded as infection and inflammatory parameters that are jointly elevated, these results illustrate that this is not always the case. CRP levels can reach very high levels in autoinflammatory diseases such as vasculitis and systemic juvenile idiopathic arthritis, while leukocyte count may be normal or only moderately elevated (29). Likewise, leukopenia instead of leukocytosis is sometimes seen in patients with sepsis or systemic lupus erythematosus (30, 31). In autoimmune disease and immunodeficiency, higher erythrocyte sedimentation rates are seen with lower levels of CRP. The different ways in which CRP and leukocyte count are associated with disease activity in different types of diseases likely explain the discrepancy between a positive association for CRP and negative association for leukocyte count in finding a focus of fever.

Aside from CRP level and leukocyte count, no other clinical or demographic factors were significantly associated with FDG-PET/CT outcome. Therefore, it remains challenging to prospectively identify those patients with FUO in whom it would be more or less likely to find a cause of fever with FDG-PET/CT.

In the study by Jasper et al., CRP, neutrophilic granulocytes, and thrombocytes correlated significantly with “positive” FDG-PET or FDG-PET/CT results. However, the definition of “positive scans” also included all scans with non-specific FDG uptake (such as elevated FDG uptake in the bone marrow, which is common in fever), and indeed, 49% of all positive scans were retrospectively regarded as “not helpful” (32). Because stand-alone FDG-PET results were also included, it is also unclear which final diagnoses were based on FDG-PET and which were based on FDG-PET/CT.

In the study by Blokhuis et al., CRP level was not associated with a true positive focus of fever on FDG-PET(/CT), which might be due to the low number of included patients. Also, by maintaining very strict criteria for FUO, it is questionable how well their study results can be extrapolated to the whole population of children with FUO (33).

Our study has some limitations. First, due to its retrospective design, there may have been selection bias. In most children with FUO, conventional diagnostics such as radiography, ultrasonography, or routine blood tests were performed before FDG-PET/CT. Only when these tests fail to identify a definite cause of fever, children might be scheduled for FDG-PET/CT. Therefore, the results of this study may not apply to all children with FUO. Second, the diagnoses at hospital discharge and results from patient follow-up were used as reference standard for FDG-PET/CT results. Although all diagnostic test results, clinical signs, response to treatment, and clinical follow-up were considered in the reference diagnosis, the diagnosis at hospital discharge was partly based on FDG-PET/CT itself and therefore might have caused verification bias.

## Conclusion

FDG-PET/CT is a valuable diagnostic tool in the evaluation of children with FUO, since it may detect a true underlying cause in almost half (48%) of all cases where none was found otherwise. It allows full-body evaluation in patients without disease-specific symptoms on one examination. In 58 out of 110 patients (53%), treatment modifications were made after FDG-PET/CT, and a new type of medication (e.g., antibiotics, immune suppression, chemotherapy) was started in 16 out of 53 children with true positive FDG-PET/CT findings. CRP and leukocyte count were significantly associated with FDG-PET/CT results, which may contribute to a priori assessment on the outcome of FDG-PET/CT. Future research could be aimed at evaluating more patient-specific factors to prospectively estimate the added value of FDG-PET/CT in children with FUO.
